# Technical application for inspection sampling for repairable systems in an economic system

**DOI:** 10.1186/s40064-016-3605-3

**Published:** 2016-11-10

**Authors:** Emmanuel Hagenimana, Song Lixin, Patrick Kandege

**Affiliations:** Dalian University of Technology, School of Mathematical Sciences, Dalian, 116023 China

**Keywords:** Acceptance test, Repairable systems, Consumer and producer, Economic parameters, Admissible strategies, Inspection sampling, Burn-in

## Abstract

In this article we develop a model for determining the appropriate level of inspection sampling for any manufacturing process. The model is useful for manufacturers, who naturally are concerned with profits and therefore with minimizing the cost of production. The model design aims to reduce total manufacturing cost and has general applicability to various manufacturing operations. The model considers the interests of consumers, who wish to minimize the cost of production while simultaneously ensuring the final product is of high quality. The cost parameters for production, the acceptance test, and admissible strategy are applied in the model. The cost components are formulated along with the minimization of the expected cost, and we used the repairable systems to guarantee the maintenance and sustainability of the economic system. We also discuss the assumptions and their appropriateness, as well as the application of the model to burn-in of system components.

## Background

The performance of the economic system is measured and fine tuned using inspection sampling. As discussed in Hamaker (1980), Hamaker (1951), to maintain a desirable level of product performance, the product value is based not only on characteristic designs but also on whether it performs to the associated specifications (Roeloffs 1967; Anscombe 1967). Although the product can be appreciated or accepted by customers according to fixed specifications as indicated above, some organizations use the test to verify the acceptance of their products in different markets.

In the case considered here, consumers and producers reach an agreement on the price of a product before it reaches the market or is delivered. This procedure helps manufacturers and their clients in that the former has the opportunity to produce more goods of a higher quality, while the latter can buy the product safe in the knowledge that it will perform as desired. Different researchers such as Kaplan and Strömberg (2004), Anderhub et al. (2002), Fehr and Gächter (2007) argue that the procedure helps producers invest enough capital in product quality.

Market competition offers advantages to both manufacturers and consumers, and forces manufacturers to communicate with customers who want quality goods at a reasonable price. While producers seek to attract customers to buy their products, customers are looking for quality products at the lowest price. Therefore, market competition benefits consumers through lower prices and improved quality of goods. For more details of how market competition benefits manufacturers and consumers refer to Mills et al. (2016) and Acharya and Lambrecht (2015).

As discussed by Berger and Udell (1998), Loss and Renucci (2012) the global investment economy depends entirely on private domestic funds, something well known to many researchers. Also, Stantcheva (2014) reveals that investments are economically significant, being a delay in wealth consumption as wealth is instead used for the manufacture of other products and for services related to the manufacturing process. Examples of investments include a factory manufacturing construction equipment, a construction business, or any company involved in production.

We often see references in the literature to investment in the organization, yet few researchers mention gross private household investment. The literature on investment discusses the financial investment of cash so as to generate income. However, it is also possible to expand the value of economic expertise or utilize the term investment to characterize all actions related to capital investment that utilizes savings, a range of activity known as financial investment savings. Interested readers can refer to Duncan (1956), van der Waerden (1960) for more details.

In this article, to test some interesting arguments such as those in Hill (1960), Singh (1966), we analyze insurance payments, and consider the acceptance test such that the results also incorporate detailed study of the mechanism of the technics in van der Waerden (1960). From the perspectives of both consumers and manufacturers, the right product will likely depend on the cost of the materials involved in its production. Consequently, test control requirements are very high. The minimum price of the product is constant and dependent on the products effectiveness or efficiency. The exchange of a defective product for a fully functional one is possible only with the payment of insurance.

The producer receives a premium if they can improve the cost of the efficient product. From Anderhub et al. (2002), Murthy and Asgharizadeh (1999) a contract where insurance is paid against the return of a defective product achieves harmony between customer and producer, which implies that the acceptance test is right, and also confirms the good quality of the product.

The study by Acemoglu et al. (2008) reveals that product quality is subjective. That is, the product characteristics that cause users to attribute a high value to a product depend on many factors. Also, the study by Flehinger and Miller (1964) envisages that the different levels of a products quality characteristics should result in various levels of consumer satisfaction.

The procedures involved in examining product quality are also discussed in Flehinger and Miller (1964). This study deals with the situation where the agreement or contract is not satisfied, but process tests are required and associated with the payment of insurance against all test outcomes. Based on the local economic situation, the manufacturer must provide just enough to maximize the product improvement. In this case, all parties benefit from product quality. Everyone must be party to a contract that helps all parties obtain profits easily.

The purpose of this article is to properly understand the relationship between customer and producer with regard to product characteristics, and the role of acceptance inspection in the economic system, something also dealt with in Acemoglu and Verdier (2000), Koch and Peyrache (2011). To achieve these objectives, we use the combination of principles and methods used in the acceptance inspection system. To improve our results, we also consider the implications of a repairable system, and so obtain a good understanding of the intervals of the product lifetime and product repair time under such a system. In this case, the customer and producer guarantee the quality and price of the goods.

The remainder of this article is organized as follows: In “A note on assumptions” section, we consider some assumptions that allow us to contribute to the main proofs provided in “Main proofs” section. In “Repairable system introduction” section, we introduce the implications of a repairable system, specifically in relation to how such a system can serve as a useful aid in model construction. In “Main proofs” section, we provide the main proofs that show how the set of admissible strategies becomes exhausted for decreasing. In “Numerical examples for clarification” section, we consider an example that clarifies aspects of our paper. In “Application to burn-in” section we discuss the application of burn-in. Finally in “Conclusion” section, we present conclusions and acknowledgments.

## A note on assumptions

In this article, we use the following assumptions in solving the problem below

$H_0$: Let $\mu$ be the composite of vectors $\mu _1, \mu _2, \ldots ., \mu _n$, that describes the *n* product parameters that collectively are known as product quality. Also, we assume that for the system state, the product parameters determine the rate of failure and the mean interval of repair. The system state increases the number of components. This implies that the proportions of the product parameters are wrong. That the proportions are wrong is supported by the acceptance test, showing that the value of the product parameters is assured.

$H_1$: We consider the function $\Theta (\mu )$ of the given parameters $\mu _i$. This function represents the motivation of the customer as the given functions increase monotonically.

$H_2$: The fundamental parameter vectors $\mu _0 ={\mu _{10}, \mu _{20}, \ldots ,\mu _{n0}}$ characterize the improvement of product quality in the case where the producer has not taken it into consideration. Therefore, by considering $\Phi (\mu )$ which is measured as a cost continuous function for the given parameters $\mu _i < \mu _{i0}$.

Because $\Phi (\mu _0)=0,$ the product improvement can help us to decrease or minimize the values of $\mu _i$ through $\mu _{i0}$. This implies that $\Theta (\mu )$ and $\Phi (\mu )$ are assigned to customers and producers.

$H_3$: Let $\tau = {\tau _1, \tau _2,\ldots \ldots ,\tau _m}$ be the parameter set that defines the test procedure with the following outcomes $\alpha _1, \alpha _2,\ldots \ldots$. From this, the probability is calculated as the function of $\mu$ and $\tau$, which is expressed as

1 $$P\{\alpha _i\mid \mu ,\tau \}$$

in correspondence with the outcome set of the given payment insurance denoted by $\delta = \delta _0, \delta _1,\ldots \ldots \ldots$. Therefore the insurance $\delta _i$ is paid according to the outcome of strategy $\alpha _i$, which determines the payment schedule and test procedure. The set of $\{\tau ,\delta \}\}$ is defined as the strategy parameters where the strategy class is taken into account by the time interval.

$H_4$: It is also assumed that a test of cost outcomes depends on testing procedure. Thus, this originates from the consumer or producer or both simultaneously. We also let $\xi _\beta (\tau )$ and $\xi _\gamma (\tau )$ denote the producer and consumer shares associated with the parameters, respectively.

$H_5$: If a new member joins either the customer or producer group and by considering the class of strategies given in assumption $H_3$, let us denote $\Upsilon _\beta$ and $\Upsilon _\gamma$ as the producer and consumer profits, respectively. Therefore from the above assumptions and the relation between $1, \Upsilon _\beta$ and $\Upsilon _\gamma$ can be expressed with regard to product parameters $\mu _i$ as well as strategy $\delta$ or $\delta _i$. Then we obtain the following equations;

2 $$\Upsilon _\beta (\mu ,\tau ,\delta )= \sum _{i=0}^\infty \delta P\{\alpha \mid \mu ,\tau \}-\Theta (\mu )-\xi _\beta (\tau )$$

3 $$\Upsilon _\gamma (\mu ,\tau ,\delta )= \Theta (\mu _0)-\Theta (\mu )-\sum _{i=0}^\infty \delta _i P\{\alpha \mid \mu ,\tau \}-\xi _\gamma (\tau )$$

Thus, the term producer profit refers to the payment of insurance where both the share of the insurance cost and the improvement to the quality investment are minimal. Similarly, we look at consumer profit as the prevention of an increasing loss in quality improvement on $\mu _0$ where investment payments are shared and costs are minimized.

To maximize profit, we assume that any given strategy $\Upsilon _\beta (\tau )$ must have a maximum strategy $\mu _i\le \mu _{i0}$; we achieve this by increasing the capital. It is known that the investment payment and the producer are tested enough to calculate the product of the given parameters $\mu _1^\star ,\ldots \ldots ..,\mu _n^\star$ that have been formulated and described as the function of the parameter strategy.

If we express or replace those parameters in the above relations (2) and (3), we get:

$$\Upsilon _\beta ^\star (\tau ,\delta )= \Upsilon _\beta (\mu ^\star ,\tau ,\delta )$$

and

$$\Upsilon _\gamma ^\star (\tau ,\delta )=\Upsilon _\gamma (\mu ^\star ,\tau ,\delta )$$

Therefore, by considering the assumption given in $H_5$, there exists a mapping between $\Upsilon _\beta ^\star , \Upsilon _\gamma ^\star$ and investment strategy that is a uniqueness application for ($\Upsilon _\beta ^\star , \Upsilon _\gamma ^\star$) given that $\Upsilon _\beta ^\star >0$ and $\Upsilon _\gamma ^\star >0$, respectively.

From this, we cannot find any investment strategy where there exists a unique application point in $(\Upsilon _\beta ^{\prime},\Upsilon _\beta ^\star )$ such that $\Upsilon _\beta ^{\prime} >\Upsilon _\beta ^\star$ and $\Upsilon _\gamma ^{\prime}\star >\Upsilon _\gamma ^\star$ or $\Upsilon _\gamma ^{\prime}\ge \Upsilon _\gamma ^\star$ and $\Upsilon _\beta ^{\prime}\ge \Upsilon _\beta ^\star$.

It is observed that the sum or $\Upsilon _\beta ^\star$ and $\Upsilon _\gamma ^\star$ has a high maximum value given by all investment strategies with equal value of $\Upsilon _\beta ^\star$. Therefore, from the relations given above in (2) and (3), we get:

$$\Upsilon _\beta (\mu ^\star ,\tau ,\delta )+\Upsilon _\gamma (\mu ^\star ,\tau ,\delta )=\Theta (\mu _0)-\Phi (\mu ^\star ) -\xi _\beta (\tau )-\xi _\gamma (\tau ).$$

Where the terms of the above equation that depend on $\mu ^\star$ do not explicitly depend on the strategy parameters, and the other two terms with the same relation depend on the given test procedure. Thus, the investment payment terms $\delta _i$ are not considered anywhere. Therefore, the $(\tau ,\delta )$ is the only unique admissible strategy, where the required expectations are shown below:

$G_0$: We may find the value of $\mu ^\star \ne \mu _0$ such that the value of

$$\Theta (\mu _0)-\Theta (\mu )-\Phi (\mu )$$

is maximized for $\mu _i\le \mu _{i0}$. The above condition is compulsory with regard to the adoption of any admissible strategy.

$G_1$: It is also assumed that the term $\Upsilon _\beta (\mu ,\tau ,\delta )$ is maximized for $\mu ^\star$ where $\mu _i\le \mu _{i0}$.

$G_2$: It is proved that for the above terms $\Upsilon _\beta (\mu ^\star ,\tau ,\delta )>0$ and $\Upsilon _\gamma (\mu ^\star ,\tau ,\delta )>0$, respectively.

$G_3$: We cannot find any other strategy point $\{\tau ^{\prime},\delta ^{\prime}\}$ where the conditions given in $G_1$ and $G_2$ fulfil the necessary information such that

$$\Upsilon _\beta (\mu ^\star ,\tau ^{\prime},\delta ^{\prime})= \Upsilon _\beta (\mu ^\star ,\tau ,\delta )$$

and

$$\xi _\beta (\tau ^{\prime})+\xi _\gamma (\tau ^{\prime})\le \xi _\beta (\tau )+\xi _\gamma (\tau )$$

are required.

Therefore, we consider the systematic strategy to determine the following admissible strategy sets: $K_0$: Consider $\mu ^\star =\mu _0$, then the term

$$\Theta (\mu _0)-\Theta (\mu )-\Phi (\mu )$$

is maximized by the value of $\mu$, where $\mu ^\star$ is determined. From this assumption, we conclude that the admissible strategy no longer exists. Therefore, according to the above assumptions no profitable strategies should exist due to the customer and producer being fully closed or bounded by

$$\Theta (\mu _0)-\Theta (\mu ^\star )-\Phi (\mu ^\star ),$$

which is well verified.

$K_1$: Contradicting the assumption given in $K_0$, let us suppose that $\mu ^\star \ne \mu _0$. It is induced that the term $\Upsilon _\beta (\mu ,\tau ,\delta )$ is highly maximized by the points strategy $\mu ^\star$, specifically in the specified domain of $\mu _i\le \mu _{i0},$ which implies that

$$\Upsilon _\beta (\mu ^\star ,\tau ,\delta )\ge \Upsilon _\beta (\mu _0,\tau ,\delta ).$$

$K_2$: By the points strategies given under the above assumption $K_1$, some terms are always considered positive, as follows

$$\Upsilon _\gamma (\mu ^\star ,\tau ,\delta )>0$$

and

$$\Upsilon _\beta (\mu ^\star ,\tau ,\delta )>0,$$

respectively.

$K_3$: Similarly, using the points strategies given in the above assumption $K_2$, the term

$$\Upsilon _\beta (\mu ^\star ,\tau ,\delta )$$

has the same value as $\Upsilon _\beta$, where we must take the value that will minimize

$$\xi _\beta (\tau )+\xi _\gamma (\tau ).$$

Therefore, there is a relationship between every value given in

$$\Upsilon _\beta (\mu ^\star ,\tau ,\delta )$$

and some of the strategies undertaken in condition $K_2$. Those are known as admissible strategies.

Hence, the focus of discussion is on why the acceptance test is most attractive to both sides in any competitive market. It is observed that the high investment decreases the product parameters at point $\mu$, and from this we can identify the point where both clients and manufacturers obtain profit, given by

$$\Theta (\mu _0)-\Theta (\mu )-\Phi (\mu ).$$

If the profit point is fit, the investment is $\Phi (\mu ^\star )$ such that the profit maximizes the strategy point $\mu ^\star$ where $\mu ^\star$. If the payment for insurance at a specified time is given by $\Phi (\mu ^\star )$, then the investment provided by

$$\Theta (\mu _0)-\Theta (\mu ^\star )$$

is established. To meet the conditions of the agreement with two parties, the producer may require some engagement via a contract; then the producer will maximize the expected profit by making the product parameters take the same value as $\mu ^\star$. Importantly, the goal of the acceptance test is to motivate the producer to maximize the profit given by $\mu ^\star$.

## Repairable system introduction

Most maintenance models consider comprehensive support where a system becomes as good as new after each maintenance action, as detailed in Duncan (1956), Endrenyi et al. (1998), Hagenimana et al. (2016). However, in reality, system performance deteriorates over time, which is why we investigate the performance of a system that is subject to imperfect repair, something also discussed in Scarf (1997). We present two cases, namely maintenance by repair and replacement, and maintenance by probabilistic repair and replacement. The objective is to assess the systems long-term behavior by deriving clues related to the expressions of its operational probability behavior.

In our case, the repairable system is applied to the economic system. To guarantee the systems sustainability, we use mechanical components or processing equipment such that experiments can be performed and the computational results given by financial parameters. For further details refer to Percy and Kobbacy (2000), Kobbacy and Murthy (2008).

Based on this, we establish some assumptions of a repairable system that are helpful in our proofs.

$M_0$: We assume that the distribution of mean $\mu \tau$ represents the number of equipment or system component failures that occur during time $\tau$. In this case, we consider only the lifetime of the system and neglect the repair time and $\mu$ gives the essential characteristics of the product material.

$M_1$: We let the given number of system failures be determined by the lifetime of the system machine, which is proportional to the expected loss of consumers, that is:

$$\Theta (\mu )=\xi _fL\mu .$$

$M_2$: We let the initial rates $\mu _0$ originate from the decreasing failure rate $\mu$ due to the amount spent by the producer, which is explained by the following relation

$$\Phi (\mu )= \xi log\left(\frac{\mu _0}{\mu }\right).$$

It is revealed that the total sum of money spent increases while the rate of change in improvement decreases.

$M_2$: We assume that the number of systems failures at any given time interval $\tau$ is 0 and that the system lifetime includes the procedures involved in the acceptance test. We also consider that the number of tests is represented by $\alpha _i$ as outcomes of a trial and that (*i*) denotes the number of failures. Finally, we assume that $\delta _i$ gives the insurance payment for all *i*.

$M_3$: We expect that the delay time to the deficiency follow the same probability density function denoted $\gamma (\eta )$ with cumulative distribution function indicated as $\Gamma (\eta )$

$M_4$: Correction of repairs at failure are taken to be minimal repairs which bring the material equipment to become as good as before.

$M_5$: The repairs at any given inspection are also considered as minimal such that they can always fix the deficiency and make the equipment materials to become good as it was before conditions. Therefore, under the assumption given to perfects inspection together with minimal repairs at inspections we establish the following:

4 $$E(\Lambda _{\gamma }(\tau _{1}))= \int _{0}^{\tau _{1}}\sigma \Gamma (\eta ){\mathrm {d}}\eta$$

5 $$E(\Lambda _{\varsigma }(\tau _{1}))= \int _{0}^{\tau _{1}}\sigma (1-\gamma (\eta ){\mathrm {d}}\eta$$

such that $E(\Lambda _{\gamma }(\tau _{1}))$ with $E(\Lambda _{\varsigma }(\tau _{1}))$ which are noticed as the number expected to the failure in interval of $(0,\tau _{1})$ and the number expected to deficiency product material at time $\tau _{1}$ respectively for each time of inspection interval are tested to be same. Given the reliability function and using the property of the poisson process we get

6 $$\chi _{1}={no \ failure \ in \ (0,\tau _{1})}=exp[-E(\Lambda _{\gamma }(\tau _{1}))]$$

where $\chi$ is assigned to the reliability level of product material. This is matched to the needed reliability which satisfies $\chi _{1}>\chi$.

$M_6$: The test interval distance is directly proportional to test cost, which is determined by the customer and the test procedures.

Therefore, we have,

$$\xi _\beta =\xi _\beta \tau$$

and

$$\xi _\gamma =\xi _\gamma \tau$$

such that, $\xi _\beta$ and $\xi _\gamma$ are the cost of product for every unit time, respectively.

From the above conditions and assumptions combined with the above relation in (2) and (3), we obtain the following expressions of profit;

7 $$\Upsilon _\beta (\mu ,\tau ,\delta )= -\xi \log \frac{\mu _0}{\mu }-\xi _\beta \tau +e^{-\mu \tau } \sum _{i=0}^\infty \delta _i\frac{(\mu \tau )^i}{(i)!}$$

8 $$\Upsilon _\gamma (\mu ,\tau ,\delta )= \xi _fL(\mu _0-\mu )-\xi _\gamma \tau -e^{-\mu \tau } \sum _{i=0}^\infty \delta _i\frac{(\mu \tau )^i}{(i)!}$$

The combination of the Eqs. (7) and (8) gives the following,

9 $$\Upsilon _\beta (\mu ,\tau ,\delta )+\Upsilon _\gamma (\mu ,\tau ,\delta ) =\xi _fL(\mu _0-\mu )-\xi \log \frac{\mu _0}{\mu }-(\xi _\beta +\xi _\gamma )\tau$$

The exact value that maximizes joint profit is obtained by differentiating the equation above with respect to $\mu$, such that is:

$$\mu ^\star =\frac{\xi }{\xi _fL}.$$

From the above we can see that $\mu _0>\mu ^\star$, which implies that

$$\mu ^\star \xi _fL=\gamma$$

$$\Rightarrow$$

$$\frac{\mu ^\star \xi _fL}{\gamma }=1$$

$$\Rightarrow$$

$$\frac{\mu ^\star \xi _fL}{\gamma }>1.$$

This matches the characteristic definition of admissible strategies.

Considering the previous assumptions, the necessary and sufficient conditions for the acceptable procedures are summarized here. Accordingly, we have two conditions as follows:

$$C_1$$: $$\delta _0=\delta {'}, \delta _i=0$$, where $$1\le i$$

$$C_2$$: $$\delta _0=\delta _0^{\prime}, \delta _1=\delta _1^{\prime}$$, $$\delta _i=0$$, where $$2\le i$$

This implies that the insurance payment is treated using the assumptions of $C_1$. This case is used to show that there is no failure of the given interval test, and the case based on the assumptions of $C_2$ is also used, thus satisfying the small insurance payment and failure in the range test.

From the assumptions given in $C_1$, the following equations (6) and (7) become;

10 $$\Upsilon _\beta (\mu ,\tau ^{\prime},\delta ^{\prime})= -\xi \log \frac{\mu _0}{\mu }-\xi _\beta \tau ^{\prime}+\delta ^{\prime} e^{-\mu \tau ^{\prime}}$$

11 $$\Upsilon _\gamma (\mu ,\tau ^{\prime},\delta ^{\prime})= \xi _fL (\mu _0-\mu )-\xi _\gamma \tau ^{\prime}-\delta ^{\prime} e^{-\mu \tau ^{\prime}}$$

respectively. The necessary and sufficient condition that equation (10) is maximized by $\mu ^\star$ is given below;

12 $$\delta ^{\prime}(\tau ^{\prime})=\frac{\xi e^{\mu \tau ^{\prime}}}{\mu ^\star \tau ^{\prime}}$$

where $\tau ^{\prime}$ is bounded in the time interval $\{\tau _1^{\prime},\tau _2^{\prime}\}$. The bounds are known as the minimum and maximum values of $\tau ^{\prime}$, respectively. Therefore the minimum $\tau _1^{\prime}$ of $\tau ^{\prime}$ is satisfied by the relation given below in (13).

13 $$\Upsilon _\gamma (\mu ^\star ,\tau ^{\prime},\delta ^{\prime}(\tau ^{\prime}))= \xi _fL(\mu _0-\mu ^\star )-\xi _\gamma \tau ^{\prime}-\frac{\xi }{\mu ^\star \tau ^{\prime}}=0.$$

While $\tau _2$ fulfils the equations below defined as

$$\Upsilon _\beta (\mu ^\star ,\tau ^{\prime},\delta ^{\prime}(\tau ^{\prime})) \ge \Upsilon _\beta (\mu _0,\tau ^{\prime},\delta ^{\prime}(\tau ^{\prime})).$$

Therefore,

14 $$\log \frac{\mu ^\star }{\mu }-\frac{1}{\mu ^\star \tau ^{\prime}} \{1-e^{(\mu _0-\mu ^\star )\tau ^{\prime}}\}\ge 0$$

15 $$\Upsilon _\beta (\mu ^\star ,\tau ^{\prime},\delta ^{\prime}(\tau ^{\prime}))= -\xi \log \frac{\mu _0}{\mu ^\star }- \xi _\beta \tau ^{\prime} + \frac{\xi }{\mu ^\star \tau ^{\prime}}\ge 0$$

By taking the derivation in the above relation given in (13) with respect to $\tau ^{\prime}$, we obtain

16 $$\frac{d\Upsilon _\gamma (\mu ^\star ,\tau ^{\prime}, \delta ^{\prime}(\tau ^{\prime}))}{d\tau ^{\prime}}= -\xi _\gamma + \frac{\xi }{\mu ^\star \tau ^{{\prime}^2}}\ge 0$$

From the given time interval bound by$\{\tau _1^{\prime},\tau _2^{\prime}\}\tau ^{\prime}$ it is implied that the assumptions given in $C_1$ are satisfied with all $\tau _1^{\prime}< \tau _2^{\prime}$, which implies that

$$\Upsilon _\beta (\mu ^\star ,\tau ^{\prime},\delta ^{\prime}(\tau ^{\prime}))$$

and

$$\Upsilon _\gamma (\mu ^\star ,\tau ^{\prime},\delta ^{\prime}(\tau ^{\prime}))$$

increase and decrease, respectively, with $\tau ^{\prime}$. It is proved that if the values of $\tau _2^{\prime}$ are established in the above relations (14) and (15), every admissible strategy will be controlled by the case given in $C_1$. Restated, if the interval of $\tau$ is approximated by the relation (14), we obtain the following relation

$$\Upsilon _\beta (\mu ^\star ,\tau ^{\prime},\delta ^{\prime} (\tau ^{\prime}))>\Upsilon _\gamma (\mu ^\star ,\tau ^{\prime},\delta ^{\prime}(\tau ^{\prime}))$$

such that $\tau ^{\prime}>\tau _2^{\prime}$, which is classified according to the assumption that $C_2$. Then we use the strategies given in assumption $C_2$. By the relation (8) given above, we obtain

17 $$\Upsilon _\beta (\mu ,\tau ^{\prime},\delta _0^{\prime}, \delta _1^{\prime})= -\xi \log \frac{\mu _0}{\mu }-\xi _\beta \tau ^{\prime} +\delta _0^{\prime} e^{-\mu \tau ^{\prime}} + \delta _1^{\prime} e^{-\mu \tau ^{\prime}}$$

and

18 $$\Upsilon _\gamma (\mu ,\tau ^{\prime},\delta _0^{\prime},\delta _1^{\prime})= \xi _fL(\mu _0-\mu ) -\xi _\gamma \tau ^{\prime} -\delta _0^{\prime} e^{-\mu \tau ^{\prime}}-\delta _1^{\prime}\mu \tau ^{\prime} e^{-\mu \tau ^{\prime}}$$

The above relation of (17) can be maximized at any given point of $\mu ^\star$ in the form

19 $$\delta _0(\tau )= \frac{\xi e^{\mu ^\star \tau ^{\prime}}}{\mu ^\star \tau ^{\prime}}+ \delta _1^{\prime}(1-\mu ^\star \tau ^{\prime})$$

Let us consider the following inequality defined as

$$\Upsilon _\beta (\mu ^\star ,\tau ^{\prime},\delta _0^{\prime}, \delta _1^{\prime})\ge \Upsilon _\beta (\mu _0,\tau ^{\prime},\delta _0^{\prime},\delta _1^{\prime}).$$

From this inequality we can obtain the following inequality described by the following relation

20 $$\delta _1\ge \frac{\xi e^{\mu ^\star \tau ^{\prime}}}{\Gamma _2(\mu _0-\mu ^ \star )}\left[\log \frac{\mu _0}{\mu ^\star }-\frac{1}{\mu ^\star \tau ^{\prime}}\left\{1-e^{-(\mu _0-\mu ^\star )\tau ^{\prime}}\right\}\right].$$

where $\Upsilon _\gamma (\mu ^\star ,\tau ^{\prime},\delta _0^{\prime},\delta _1^{\prime})$ is effectively maximized for every value of $\tau ^{\prime}$. The above relation to (20) may be used to find the values of $\delta _1^{\prime}(\tau ^{\prime})$ and $\delta _0^{\prime}(\tau ^{\prime})$, respectively. Just as above, the admissible strategies defined in the assumed condition $(C_2)$ of bounded $\tau$ given in the interval $\tau _3^{\prime},\tau _4^{\prime}$ are such that $\tau _2^{\prime}\le \tau _3^{\prime}$ and we obtain the following results

21 $$\Upsilon _\gamma (\mu ^\star ,\tau _3^{\prime},\delta _0^{\prime} (\tau _3^{\prime}),\delta _1^{\prime}(\tau _3^{\prime}))=\Upsilon _\gamma (\mu ^\star ,\tau _2^{\prime},\delta ^{\prime}(\tau _2^{\prime})).$$

We know that, $\tau _4^{\prime}$ is the highest value of $\tau ^{\prime}$ in the given interval $(\tau _3^{\prime},\tau _4^{\prime})$ of $\tau ^{\prime}$, which is expressed by the following relation,

22 $$\Upsilon _\beta (\mu ^\star ,\tau ^{\prime},\delta _0^{\prime}(\tau ^{\prime}), \delta _1^{\prime}(\tau ^{\prime}) \ge 0$$

Applying the derivative with respect to $\tau ^{\prime}$ in the above (22) relation, we get the following,

23 $$\frac{d[\Upsilon _\gamma (\mu ^\star ,\tau ^{\prime},\delta _0^{\prime} (\tau ^{\prime}),\delta _1^{\prime}(\tau ^{\prime})]}{d\tau ^{\prime}}\ge 0$$

## Main proofs

From the assumptions given in $C_2,$ which are clearly defined, where $\tau _4>\tau _3$, then by using different techniques, we have to prove that the conditions defined in $C_1$ and $C_2$ are admissible strategies. To verify this, we must use the necessary and sufficient conditions determined by repairable systems for admissible strategies. $\Upsilon _\beta (\mu ,\tau ,\delta )$ is maximized at the admissible strategy point $\mu ^\star$ such that,

$$\frac{d\Upsilon _\beta (\mu ,\tau ,\delta )}{d\mu }|_{\mu =\mu ^\star }=0$$

This is identical to writing,

24 $$\delta _0=\frac{\xi e^{\mu ^\star \tau }}{\mu ^\star \tau }+\sum _{i=0}^\infty \delta _{i+1}\frac{(\mu ^\star \tau )^i}{(i)!}\left[1-\frac{\mu ^\star \tau }{i+1}\right].$$

Therefore, we have,

25 $$\begin{aligned} \Upsilon _\beta (\mu ,\tau ,\delta )&= -\xi \log \frac{\mu _0}{\mu }-\xi _\beta \tau +\frac{\xi e^{-(\mu -\mu ^\star )\tau }}{\mu ^\star \tau }+e^{-\mu \tau }\sum _{i=0}^\infty \delta _{i+1}\frac{(\mu ^\star \tau )^i}{(i)!}\nonumber \\&\quad-\frac{(\mu ^\star \tau )^{i+1}}{(i+1)!}+\frac{(\mu ^\tau )^{i+1}}{(i+1)!}\end{aligned}$$

We have seen that

$$\Upsilon _\beta (\mu ^\star ,\tau ^{\prime},\delta _0^{\prime}, \delta _1^{\prime})\ge \Upsilon _\beta (\mu _0,\tau ^{\prime},\delta _0^{\prime},\delta _1^{\prime}).$$

Similarly, we have

$$\Upsilon _\beta (\mu ^\star ,\tau ,\delta )\ge \Upsilon _\beta (\mu _0,\tau ,\delta )$$

which is derived in the above relation (20). Therefore, we get the following,

26 $$\begin{aligned}\delta _1&\ge \frac{\xi e^{\mu ^\star \tau }}{\Gamma _2[(\mu _0-\mu ^\star )]}\left[\log \frac{\mu _0}{\mu ^\star }-\frac{1}{\mu ^\star \tau }\{1-e^{-(\mu _0-\mu ^\star )\tau }\}\right]-\frac{1}{\Gamma _2[(\mu _0-\mu ^\star )]}\nonumber \\&\quad-\sum _{i=0}^\infty \delta _{i+1}\frac{(\mu ^\star \tau )^i}{(i)!}\left[1- e^{-(\mu _0-\mu ^\star )\tau }\left\{1+\frac{\mu ^\star \tau }{i+1}(\frac{\mu _0^{i+1}}{\mu ^{\star i+1}})\right\}\right]\end{aligned}$$

From the assumption given above in $C_1,$ $\delta _0=\delta ^{\prime},$ $\delta _i=0,$ $i\ge 1$, we must check for admissible strategies. Using the relation given above in (24) we get the following,

27 $$\delta ^{\prime}(\tau ^{\prime})=\frac{\xi e^{\mu ^\star \tau ^{\prime}}}{\mu ^\star \tau ^{\prime}}$$

Therefore, from the equation given in (25), we get the following

28 $$-\log \frac{\mu _0}{\mu }+\frac{1}{\mu ^\star \tau ^{\prime}}\{1-e^{-(\mu _0-\mu ^)\tau ^{\prime}}\}\ge 0$$

From the above equation given in (27), an upper bound fixed on $\tau ^{\prime}$ is observed. Therefore the producer and consumer are determined by the following

29 $$\Upsilon _\beta (\mu ^\star ,\tau ^{\prime},\delta ^{\prime})=\xi \log \frac{\mu _0}{\mu ^\star }-\xi _\gamma \tau ^{\prime} +\frac{\xi }{\mu ^\star \tau ^{\prime}}> 0 \ \ \ as \ \ \ \Upsilon _\beta (\mu ^\star ,\tau ^{\prime},\delta ^{\prime})>0$$

Similarly we get

30 $$\Upsilon _\gamma (\mu ^\star ,\tau ^{\prime},\delta ^{\prime})=-\xi \log L(\mu _0-\mu ^\star )-\xi _\gamma \tau ^{\prime}-\frac{\xi }{\mu ^\star \tau ^{\prime}}> 0 \ \ \ as \ \ \ \Upsilon _\gamma (\mu ^\star ,\tau ^{\prime},\delta ^{\prime})> 0$$

Therefore, the above equation is given in (29), we obtain the bound interval defined on $\tau ^{\prime}$ and from that, relation (30) has a minimized value that is around $\tau$ provided that $\Upsilon _\gamma (\mu ^\star ,\tau ^{\prime},\delta ^{\prime}(\tau ^{\prime})$ and $\Upsilon _\beta (\mu ^\star ,\tau ^{\prime},\delta ^{\prime}(\tau ^{\prime})$ increase and decrease, respectively, around the point $\frac{d\Upsilon _\gamma }{d\tau ^{\prime}}$.

Hence, using the above Eq. (30), we obtain the following,

31 $$\frac{d\Upsilon _\gamma (\mu ^\star ,\tau ^{\prime},\delta ^{\prime}(\tau ^{\prime}))}{d\tau ^{\prime}}=\xi _0 + \frac{\xi }{\mu ^\star \tau ^{{'}^2}}\ge 0$$

We must verify that the given strategy point $\{\tau ^{\prime},\delta ^{\prime}\}$ defined by assumption $(C_1)$ satisfies equations (27–29) and (30) and that the admissible strategy is complete. Also, we must verify other strategy points such as $\{\tau ,\delta \},$ to prove that Eq. (24) is also satisfied. Therefore, we get the following important inequality

$$\Upsilon _\beta (\mu ^\star ,\tau ,\delta ) + \Upsilon _\gamma (\mu ^\star ,\tau ,\delta )>\Upsilon _\beta (\mu ^\star ,\tau ^{\prime},\delta ^{\prime}(\tau ^{\prime})) + \Upsilon _\gamma (\mu ^\star ,\tau ^{\prime},\delta ^{\prime}(\tau ^{\prime})),$$

this express that $\tau ^{\prime}>\tau$ such that

$$\Upsilon _\gamma (\mu ^\star ,\tau ,\delta )=\xi _fL(\mu ^\star -\mu _0) -\xi _\gamma \tau -\frac{\xi }{\mu ^\star \tau }-e^{-\mu ^\star \tau } \sum _{i=0}^\infty \delta _{i+1}\frac{(\mu ^\star \tau )^i}{(i)!} <\Upsilon _\gamma (\mu ^\star ,\tau ^{\prime},\delta ^{\prime}(\tau ^{\prime}))$$

Therefore, from the above expression we cannot find any other strategy point besides $\{\tau ^{\prime},\delta ^{\prime}(\tau ^{\prime})\}$. that corresponds to the point of maximum value to the producer $\Upsilon _\beta (\mu ^\star ,\tau ,\delta )$ where the maximum value lies in the interval $\tau ^{\prime}$.

Besides this, as the maximum value $\tau ^{\prime}$ is expressed by the Eqs. (30) or (32) as given above, it shows that all admissible strategies lie in assumption $(C_1)$, which has a greater value than the maximum given under condition $(C_1)$ because $\tau >\tau _2^{\prime}$. Furthermore, we analyze the strategy points given in assumption $(C_2)$ determined by the following

$$\delta _0=\delta _0^{\prime}, \,\,\delta _1=\delta _1^{\prime},\,\, \delta _i^{\prime}=0,\quad i\ge 2$$

which must satisfy the conditions below:

$$\Upsilon _\beta (\mu ^\star ,\tau ^{\prime},\delta _0^{\prime}(\tau ^{\prime}), \delta _1^{\prime}(\tau ^{\prime}))=\Upsilon _\beta (\mu _0,\tau ^{\prime}, \delta _0^{\prime}(\tau ^{\prime}),\delta _1^{\prime}(\tau ^{\prime})),$$

such that

32 $$\delta _1^{\prime}(\tau ^{\prime})=\frac{\xi e^{\mu ^\star \tau }}{\Gamma _2[(\mu _0-\mu ^\star )\tau ]} \left[\log \frac{\mu _0}{\mu ^\star }-\frac{1}{\mu ^\star \tau }\{1-e^{-(\mu _0-\mu ^\star )\tau }\}\right].$$

In the same way we have,

33 $$\delta _0^{\prime}(\tau ^{\prime})=\frac{\xi e^{\mu ^\star \tau ^{\prime}}}{\mu ^\star \tau ^{\prime}}+\delta _1^{\prime} (\tau ^{\prime})(1-\mu ^\star \tau ^{\prime})$$

And

34 $$\Upsilon _\gamma (\mu ^\star ,\tau ^{\prime},\delta _0^{\prime}(\tau ^{\prime}), \delta _1^{\prime}(\tau ^{\prime})) > \Upsilon _\gamma (\mu ^\star ,\tau _2^{\prime},\delta ^{\prime}(\tau _2^{\prime}))$$

35 $$\Upsilon _\beta (\mu ^\star ,\tau ^{\prime},\delta _0^{\prime}(\tau ^{\prime}),\delta _1^{\prime}(\tau ^{\prime}))> 0$$

36 $$\frac{d\Upsilon _\gamma (\mu ^\star ,\tau ^{\prime},\delta _0^{\prime}(\tau ^{\prime}),\delta _1^{\prime}(\tau ^{\prime})}{d\tau ^{\prime}}> 0$$

The relation (34) fix a minimum value on the possible range of $\tau ^{\prime}$ and relation (35),(36) adjust upper bounds on $\tau ^{\prime}$. To demonstrate that any strategy in category $C_{1}$ which fulfils conditions (32–36) is admissible, take into consideration other strategy $(\tau ,\delta )$ which assures (24) and (25) and for which

37 $$\Upsilon _\beta (\mu ^\star ,\tau ,\delta ) +\Upsilon _\gamma (\mu ^\star ,\tau ,\delta )> \Upsilon _\beta (\mu ^\star ,\tau ^{\prime},\delta _0^{\prime}(\tau ^{\prime}), \delta _1^{\prime}(\tau ^{\prime}))+\Upsilon _\gamma (\mu ^\star ,\tau ^{\prime},\delta _0^{\prime}(\tau ^{\prime}),\delta _1^{\prime}(\tau ^{\prime}))$$

We shall demonstrate that

38 $$\Upsilon _\gamma (\mu ^\star ,\tau ,\delta )> \Upsilon _\gamma (\mu ^\star ,\tau ^{\prime},\delta _0^{\prime}(\tau ^{\prime}),\delta _1^{\prime}(\tau ^{\prime}))$$

From the relation (34) it is shown that (38) is correct for all strategies given in category $C_{1}$. Then let us consider that those strategies for which $\delta _i^{\prime}> 0$ for some $i>0$. The relation (37) suggests that $\tau <\tau ^{\prime}$ and from the above relations (24) together with (26) we have

39 $$\begin{aligned}\Upsilon _\gamma (\mu ^\star ,\tau ,\delta )&\le \xi _fL(\mu _0-\mu ^\star )-\xi _\gamma \tau -\frac{\xi }{\mu ^\star \tau }-\frac{\xi }{\Gamma _2[(\mu _0-\mu ^\star )\tau ]}\nonumber \\&\quad \cdot \left[\log \frac{\mu _0}{\mu ^\star }-\frac{1}{\mu ^\star \tau }\{1-e^{-(\mu _0-\mu ^\star )\tau }\}\right]\nonumber \\&\quad -\frac{(\mu _0-\mu ^\star )\tau e^{-\mu _0\tau }}{\Gamma _2[(\mu _0-\mu ^\star )\tau ]}\sum _{i=1}^\infty \delta _{i+1}\frac{(\mu ^\star \tau )^i}{(i)!}\left[\frac{1}{i+1}\sum _{j=0}^i(\frac{\mu _0}{\mu ^\star })^j-1\right] \end{aligned}$$

But from relation (35), we have noticed that

40 $$\begin{aligned}\Upsilon _\gamma (\mu ^\star ,\tau ^{\prime},\delta _0^{\prime}(\tau ^{\prime}),\delta _2^{\prime}(\tau ^{\prime}))&= \xi _fL(\mu _0-\mu ^\star )-\xi _\gamma \tau -\frac{\xi }{\mu ^\star \tau ^{\prime}}-\frac{\xi }{\Gamma _2[(\mu _0-\mu ^\star )\tau ^{\prime}]}\nonumber \\&\quad \cdot \left[\log \frac{\mu _0}{\mu ^\star }-\frac{1}{\mu ^\star \tau ^{\prime}}\{1- e^{-(\mu _0-\mu ^\star )\tau ^{\prime}}\}\right]\nonumber \\&\quad >\xi _fL(\mu _0-\mu ^\star )-\xi _\gamma \tau -\frac{\xi }{\mu ^\star \tau }-\frac{\xi }{\Gamma _2[\mu _0-\mu ^\star )\tau ]}\left[\log \frac{\mu _0}{\mu ^\star }-\frac{1}{\mu ^\star \tau }\{1- e^{-(\mu _0-\mu ^\star )\tau }\}\right]\end{aligned}$$

The above relation (38) follows from (39) and (40) such that no other strategies in category $C_{1}$ have dominance $(\tau ^{\prime},\delta _0(\tau ^{\prime}),\delta _1^{\prime}(\tau ^{\prime}))$. Provided the upper bound on $\tau ^{\prime}$ be established by (35) or (36) it ensure that there is no admissible strategy that can have a significant value of $\Upsilon _\gamma (\mu ^\star ,\tau ,\delta )$ remarkable than that corresponding to this upper bound, so that the admissible strategies have been completely exhausted.

## Numerical examples for clarification

### *Example 1*

Let us consider an example involving the condition that a machine system with a lifetime of 10,000 for a period of 60 min costs the consumer $\pounds 300$ per failure. Additionally, the fundamental loss rate is 0.1, and if the rate of giving loss is $\mu$, then the cost of production at each failure is 35,000 log 0.1 $(\mu )$. Therefore, we consider $\xi _f=300, L=10000$, $\xi =35000, \mu =0.1, \xi _\gamma =200$ and $\xi _\beta =0$. From the above, let the calculation of $\mu ^\star$ be given by the relation

$$\mu ^\star =\frac{\xi }{\xi _fL}.$$

**Fig. 1:**
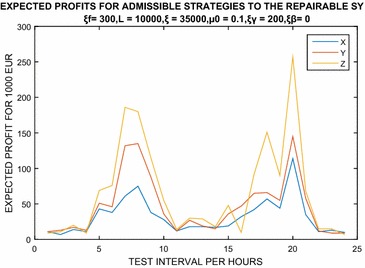
Example 1: Expected profits for admissible strategies to repairable system. We have seen that, the test range of 17.83 in period of 60 min to payment for insurance of 207,200 yields, is equivalent to the given profit to the manufacturer and clients of 13,100 EUR to each failure

The calculation yields a value of $0.0116666667\approx 0.0117$. This proves that the admissible strategies all occur under the conditions $C_1$. These conditions have an interval, ranging from 11.42 h, while $\Upsilon _\gamma =0$ ranges from 38.57 h, such that $\Upsilon ^\star _\beta =\Upsilon _\beta (\mu _0)$. This corresponds to the premium payment for no failures ranging from $\pounds 300100$ up to $\pounds 122000$, a range that has been tested as equivalent to the corresponding profit earned by manufacturers and customers. We have seen that the test range of 17.83 in a 60-min period given an insurance payment of 207,200 is equivalent to profit for the manufacturer and clients of $\pounds 13100$ per failure, as explained in the table below. We obtain the interpretation results using Matlab software as shown in Fig. 1 below. We note that $X = \Upsilon _\beta (\mu _0),$ $Y =\Upsilon _\beta ^{\star },$ and $Z =\Upsilon _\gamma ^{\star }$ denote an expected producer, and premium payments for a manufacturer and a consumer, respectively, as shown in the table below.

### *Example 2*

By this example, we consider the model that expanded early and evaluated the validity of the product materials. From this model notation, we have a significant number of cost and downtime parameters which required to be carefully taken into consideration. In this example we use three kinds of production options which help us to analyse and understand the process of the inspection sampling such as: In product option one, the producer takes out controls and repairs to correct the deficiency product materials recognized at the inspection of the respectively reasonable interval of time. The manufacturer accomplishes correction of repair to the failure up to the end of the service based on the agreement of the period.

The customer compensates cost and needs to meet a particular level of reliability and availability of the product materials. Then if the price is fixed, producer accomplishes failure of the repairs and then inspection to the system where he corrects all types of failures and deficiency found over agreement of the period without considering the extra cost to the customer. Since failure is not amended in agreement time, the producer includes punishment which gives guarantee to the producer that the client has all right to use the product materials for too long.

In the product option 2, the customer takes out inspections and repairs to the deficiency product content noticed at time of inspections on the respectively reasonable interval of time.

The manufacturer accomplishes correction of repair to failure up to the end of the service due to the agreement of deadline. Here as failure takes place, the customer addresses the problem of the producer to repair the failed product item. The producer request for payment of the debt amount for all repairs without considering extra parts and act of punishment related to the term and conditions for product item remains in failed specification time.

Hence the customer thinks reasonable to get the same level to the reliability and availability of product materials respectively.

In product option three the customer executes correction to the repairs of failures throughout of agreement period to the inspections where repair at the time of inspections of product materials at a reasonable interval of time are taken out by the producer.

Here customers consider reasonably to get the required level to the reliability and availability which is preserved. Based on the introduction to these three product options we proceed as follows,

The relation exists between some of the cost and downtime parameters must be reasonably assigned. We assume that: The extra part to the average cost is taken as $\$ 500,$ and the working time per person cost of the repair staff to the customer is $\$ 80$ per working day. The working hours per individual cost for the repair crew to the producer is $\$ 120$ per day per parson. We suppose that the failure downtime is taken as four times of inspection downtime per repair of the deficiency considered at an inspection is given as $\frac{1}{20}$th of the failure repair downtime.

The additional is made according to the inspection sampling of repair to the product materials arranged. Consequently, there are some persons and extra parts who are prepared for the work where the repair crew consists of the individuals for both producer and customers.

For the option two and three, the manufacturer only replaces the workforce cost with a profit margin (20% of the employment cost) since the customer compensates for the extra part cost. Based on the different background and efficiency, the customers pass more time on inspection and repairs materials.

We suppose that the downtime caused by the inspections and repair done under the control of the customers is three times compared to that one observed in product option one. Therefore for the product option three, the downtime for failure repair by the client to that one done by the producer is similar to that one observed in product option two.

Hence different costs are estimated by using the following formula accordingly,

41 $$\begin{aligned}C&= {\textit{number\,of\,day\,of\,persons}}\,\times \,{\textit{days \,in\,the\,work\,daily\,pay\,rate}}\nonumber \\&\quad\times (1+ {\textit{profit\,margin\,percentage\,if\,any}}) +{\textit{part\,if\,exist}}\end{aligned}$$

where *C* is known as to the production cost of the equipment materials.

From the above relation (41) producer or customer have the required information, in such that the other party has to decide the offer done based on the working time per individual on their availability.

The basic information is the estimated value of the arrival of deficiency product materials to the delay time distribution work and its parameters, and also different downtime and cost information are also considered.

Assume that the producer has the whole information and attempts all options as the following.

Product of option one finishing maintenance service with the cost of $\$ 50.00$ to the agreement period.

Product of option two, the failure is based only on maintenance and cost of $\$ 864$ per failure without the extra part’s cost.

And hence, product in option three, inspection plus repair have cost of $\$ 216$ per inspection and $\$ 43$ for deficiency product materials amended during the inspection without the extra part’s cost.

However, the inspection must be accomplished within 10 days of interval time. It was noticed that, if the customers were able to evaluate the best option for them, they should also maintain and control all information which is an obstacle to them while they only have had limited information.

Therefore customers are obliged to guess the estimated rate to the arrival of deficiency product materials. Let the parameter to the delay time distribution be assumed as exponential distribution based on the given above information:

The availability and reliability are required and then producer propose the inspection at interval time and then guess the values of the downtime information based on the cost charged to customer under the given product of option 2 and three respectively.

Assume that customer has found the workforce required to accomplish work. The present employer rate to the producer together with the market profit margin, and he may compute the down times evaluated by manufacturer using the relation above in (41). And then if down times have evaluated, it is easy to get the estimated value of $\alpha$ and $\lambda$ by also using expressions above in 6 due to the availability and reliability levels to the producer proposed in inspection sampling related to the time interval.

The computation of $\sigma$ and $\lambda$ are not taken to be exact while the significance value employed by the producer on the required availability and reliability levels. Therefore, the customer could estimate downtime, which is larger that producer’s ones. Assume that customer his determined to producer’s downtime by using the above relation given in (41) times three times to obtain his assessment values and then parameters $\lambda$ and $\sigma$ are founded.

Using the given above relation in (4) $E(\Lambda _{\gamma }(10))\le 0.8,$ is closed related to $0.8\le e^{-E(\Lambda _{\gamma }(10))}$ which has an estimated value of $E(\Lambda _{\gamma }(10))\le 0.222$.

Therefore by the given information to the expected downtime, the reliability level required with helped by the expression given in (6). We have the assessed value of $E(\Lambda _{\gamma }(10))$ that is $E(\Lambda _{\varsigma }(10))\le 0.531$.

From here, we are now able to evaluate the value of $\lambda$ and $\sigma$. $\lambda$ can be evaluated or estimated at once using the following expression,

$$\sigma =\frac{(E(\Lambda _{\gamma }(10)+E(\Lambda _{\varsigma }(10)))}{10}\le 0.07$$

while the parameter $\sigma$ is evaluated according to the relation provided in (4) or (5) with $\Gamma (\eta )$ may be taken as an exponential.

The condition that $\lambda \le 0.05,$ motivates customers to run the same model due to those two evaluated parameters. And conclude that the given product materials in option one are taken as the best to be considered on their side while the producer on his side there is no profit, only loss without even considering the inspection interval time used in his inspection sampling process.

We noticed that it is totally impossible to understand how producer can get profit which is similar to the chosen from three products options.

Therefore to obtain this, the customers have to repeat the identical model with essay and error, for getting the exact combination to the $\sigma$ with $\lambda$. It can supply logical answer which must be closed related to the producer’s estimation of $\sigma$ and $\lambda$ where they have been estimated as 0.01 and 0.01 respectively.

From the estimated parameters designed above, the customers are now able to run their model and emphasize to the inspection interval time needs to assess the agreement accepted by the product option one. Therefore the producer on his side is a pleasure to agree on the contract since he is only marginally worse off but still evaluated as better than that of product option two and three respectively.

For more details regarding numerical example of this model see Golmakani and Moakedi (2012, 2013), Murthy and Asgharizadeh (1999), Zhao et al. (2012)

## Application to burn-in

The product quality is assumed to be represented by the length of time the product provides satisfactory service and thus is closely related to product lifetime. After products are manufactured, it is suggested that they be put into operation for a particular period of time to test their quality; only products found to be of good quality are put into service, while defective products are tested and improved. For Weibull, Gamma, Exponential and Extreme values can be seen in Mi (1994), Scarf (1997). The use of *b* in this section results from the selection criterion. Assume that cost is not a consideration and we simply want to maximize mean life. Consequently, we want to determine *b* such that the mean residual life is minimized because only those items that survive with a fixed burn-in time are replaced by the given services, which are established in Watson and Wells (1961). Consequently, we want to find $b^{\star }$ such that the following statement is true,

$$\mu (b^{\star })= max_{b\ge 0}\mu (b).$$

Let the given $b^{\star }$ be the optimal burn-in time. We consider the case where *F* has a bathtub-shape curve $h(\tau )$ in the following situations:

1. $\tau _{1}=0,$ in this case, there is little or no need to burn-in, and it follows that $b^{\star }=0$. This statement is always taken as true.
2. $\tau _{2}=\infty$ and $\tau _{1}>0,$ in this situation, we are always allowed to choose $b^{\star }=\tau _{1}$.
3. $\tau _{1}=\tau _{2}=\infty ,$ *F* is known as the decreasing failure rate function, and in this case the cost should be taken into consideration.
4. $0<\tau _{1}\le \tau _{2}<\infty ,$ Thus, the value of $b^{\star }$ is equivalent to the unique change point $\tau ^{\star }$ of $\mu (\tau )$.

Accordingly, we do not need to burn-in products for a long time because of the first change point $t_{1}$ where the failure rate function *F* is decreasing.

Another application is motivated by Mi (1995). Let us consider the cost component that has a lifetime $X_{1}$ with a cumulative distribution function. Suppose that burn-in for this kind of component occurs at a given time *b*. Further suppose that the component survives the burn-in. In this case the component is allowed to pass into field operations, in contrast to the assumption above. A new component with lifetime $X_{2}$ (i.i.d) as $X_{1}$ is taken or considered for the field operation. Our target now is to find the optimal burn-in time by minimizing the mean life of the cost components, which are finally used in field operation after a long delay.

Let $\xi (b)$ be considered the lifetime of the cost component used in the field operation and consider the equation below,

$$\xi (b)=(X_{1}- b).\jmath (X_{1}> b)+ X_{2}.\jmath (X_{1}\le b).$$

Hence, the mean life of the cost component in the field operation is given by

$$E{\xi (b)}=\int _{b}^{\infty }(\tau -b){\mathrm {d}}F(\tau ) +\mu .F(b)=\int _{b}^{\infty }R(\tau ).\tau {\mathrm {d}}\tau + \mu .F(b)=R(b).[\mu (b)-\mu ]+\mu .$$

Let $F(\tau )$ exhibit a bathtub curve $h(\tau )$ without decreasing failure rate function. From the above assumptions, there exists a unique $\widetilde{b}\le \tau _{1}$ in which $\mu \widetilde{b}>\mu (b),$ $\forall b\ne \widetilde{b},\mu (b),$ is strictly increasing into $b>\widetilde{b}$. From the above result, we obtain the following

$$E{\xi (b)}<R(\widetilde{b}).[\mu (\widetilde{b})-\mu ]+\mu =E{\xi (\widetilde{b})},\quad \forall b>\widetilde{b}.$$

Therefore, $\widetilde{b}$ maximizes $E{\xi (b)}$ and must be in the given interval of $[0,\widetilde{b}]\subset [0,\tau _{1}]$.

Let us consider the situation where

$$\frac{d}{{\mathrm {d}}b}(E{\xi (b)})=\mu .R(b).\left[h(b)-\frac{1}{\mu }\right]$$

Then from the bathtub curve of $R(\tau ),$ the $b^{\star }$ maximum $E{\xi (b)}$ exists and is unique.

For more detail refer to Mi (1995), Lai and Xie (2006), Block et al. (1994) where several cost component structures are associated with burn-in as well as with the field operation in which only those cost components that survive the burn-in are considered. That is, the cost component may consist of two parts, one being incurred by the burn-in procedure and belonging to the producers, while the other is related to the field users and belongs to the consumer. Therefore, the gain or benefit is proportional to the given minimum life of the product of the field operation. Any other lifetime replacement policy is spontaneously applied (Table 1).

**Table 1:** Admissible strategies $\xi _f=300,L=10000,\xi =35000,\mu _0=0.1, \xi _\gamma =200,\xi _\beta =0$

$\tau$	$\delta$	$\Upsilon _\beta ^\star$	$\Upsilon _\beta (\mu _0)$	$\Upsilon _\gamma ^\star$	$\Upsilon _\beta ^\star +\Upsilon _\gamma ^\star$
11.42	300,100	187,500	95,800	0	187,500
15	238,200	124,800	53,100	62,000	186,800
17.83	207,200	93,100	34,800	93,100	186,200
20	189,400	74,800	25,600	111,000	185,800
25	160,600	44,800	13,200	140,000	184,800
30	141,900	24,800	7100	159,000	183,800
35	128,900	10,500	3900	172,300	182,800
38.57	122,000	2600	2600	179,500	182,100

## Conclusion

In this paper, we developed a model based on the cost of producing an effective or defective item, our aim being to minimize the production cost of that item. Minimization of production cost thus is the primary objective of this article. By associating the probabilities of generating effective or defective items with the cost of production, we can obtain the real total cost of production. The economic design of a single sampling attribute inspection plan is the purpose of the development of the cost model presented in this article. It is easy for an individual to adjust to problem solving because all the cost components are integrated into the design model. Trial and error can obtain the acceptance sampling plan that results from the lowest cost. It is then necessary to know the distribution of the entire process; we use the binomial distribution for the whole process of determining defective items along with arbitrary cost data. We observed that it is impossible to find other admissible strategies similar to the previous one, which proves the uniqueness of problem-solving strategies by using the implications of repairable systems. In this case, we neglect the repair time by considering only the lifetime of repairable systems. The numerical application results show that the test was performed in the range 17.83 over a 60-min period during which insurance was purchased at a cost of $\pounds 207200$, and the profit expected to be available for equal sharing by the customers and the manufacturer was $\pounds 13100$ for each failure. In this article we also discussed the burn-in as an application. Furthermore, inspection is necessary to safeguard system components against destruction. Future research will investigate different maintenance conditions.
